# Molecular Epidemiology of *Clostridium difficile* at a Medical Center in Taiwan: Persistence of Genetically Clustering of A^−^B^+^ Isolates and Increase of A^+^B^+^ Isolates

**DOI:** 10.1371/journal.pone.0075471

**Published:** 2013-10-07

**Authors:** Ju-Hsin Chia, Hsin-Chih Lai, Lin-Hui Su, An-Jing Kuo, Tsu-Lan Wu

**Affiliations:** 1 Department of Laboratory Medicine, Chang Gung Memorial Hospital, Linkou, Kweishan, Taoyuan, Taiwan; 2 Department of Medical Biotechnology and Laboratory Science, Chang Gung University, Kweishan, Taoyuan, Taiwan; 3 Graduate Institute of Biomedical Sciences, Chang Gung University, Kweishan, Taoyuan, Taiwan; St. Petersburg Pasteur Institute, Russian Federation

## Abstract

**Introduction:**

We investigated the changing trend of various toxigenic *Clostridium difficile* isolates at a 3 500-bed hospital in Taiwan. Genetic relatedness and antimicrobial susceptibility of toxigenic *C. difficile* isolates were also examined.

**Methods:**

A total of 110 non-repeat toxigenic *C. difficile* isolates from different patients were collected between 2002 and 2007. Characterization of the 110 toxigenic isolates was performed using agar dilution method, multilocus variable-number tandem-repeat analysis (MLVA) genotyping, *tcdC* genotyping, and toxinotyping.

**Results:**

Among the 110 toxigenic isolates studied, 70 isolates harbored *tcdA* and *tcdB* (A^+^B^+^) and 40 isolates harbored *tcdB* only (A^−^B^+^). The annual number of A^+^B^+^ isolates considerably increased over the 6-year study (*P* = 0.055). A total of 109 different MLVA genotypes were identified, in which A^+^B^+^ isolates and A^−^B^+^ isolates were differentiated into two genetic clusters with similarity of 17.6%. Twenty-four (60%) of the 40 A^−^B^+^ isolates formed a major cluster, MLVA-group 1, with a similarity of 85%. Seven (6.4%) resistant isolates were identified, including two metronidazole-resistant and five vancomycin-resistant isolates.

**Conclusions:**

This study indicated a persistence of a MLVA group 1 A^−^B^+^ isolates and an increase of A^+^B^+^ isolates with diverse MLVA types. Moreover, *C. difficile* isolates with antimicrobial resistance to metronidazole or vancomycin were found to have emerged. Continuous surveillance is warranted to understand the recent situation and control the further spread of the toxigenic *C. difficile* isolates, especially among hospitalized patients.

## Introduction

*Clostridium difficile* is an anaerobic, gram-positive, spore-forming bacillus. It is one of the most common nosocomial pathogens identified and is the primary cause of antibiotic-associated diarrhea. [1] *C. difficile*-associated disease (CDAD) encompasses diseases of a range of severity from uncomplicated mild diarrhea to toxic megacolon that can result in sepsis and even death. [1] CDAD has been an increasing problem in health care, especially because hypervirulent strains (ribotype 027, toxinotype III, and pulse-field NAP1) have emerged in North America and Europe over the past 10 years. [2], [3], [4] The pathogenicity of *C. difficile* is primarily based on the action of at least one of the two major exotoxins produced and secreted by the bacteria, i.e., toxin A (enterotoxin) and toxin B (cytotoxin), which are encoded by the *tcdA* and *tcdB* gene, respectively. [5], [6] In addition, some *C. difficile* isolates also produce a binary toxin called CDT, which is an actin-ADP-ribosylating toxin. [7] Although the pathological role of CDT in CDAD remains unclear, CDT contributes to CDAD and has been associated with increased disease severity. [8], [9].

Laboratory diagnosis of CDAD is currently achieved by isolation of toxigenic *C. difficile* isolates from stool samples and detecting the produced toxins. Several methods can be used to diagnose *C. difficile* infection. These methods included *C. difficile* culture, cell cytotoxicity assay from stool filtrates, latex agglutination for the detection of *C. difficile*-associated antigen in stools, and enzyme immunoassay for the detection of toxin A, toxin B or both from stool samples. [10], [11] Recently, to distinguish toxigenic from non-toxigenic *C. difficile* isolates, a multiplex-PCR assay simultaneously amplifying *tcdA* and *tcdB* genes was developed. [12] PCRs for the detection of binary toxin and *tcdC* gene deletion were also studied. [13] A highly sensitive real-time PCR method for the rapid detection of toxigenic *C. difficile* in stool samples had also been used for diagnosing CDAD. [14], [15], [16].

The antibiotics metronidazole and vancomycin are frequently used to treat CDAD. Oral metronidazole is the drug of choice for initial CDAD therapy because of its lower cost and concerns regarding the proliferation of vancomycin-resistant nosocomial bacteria. Vancomycin is recommended for treatment in patients with severe infection because of faster symptom resolution and a significantly lower risk of treatment failure. [17] As previous reports have indicated, *C. difficile* clinical isolates were sensitive to metronidazole or vancomycin, [18] clinical laboratories do not routinely perform antimicrobial susceptibility tests on this organism. However, up to 6.3% of toxin-producing isolates with resistance to metronidazole, and 3% with intermediate resistance to vancomycin were reported. [19] Poor outcomes of metronidazole therapy in CDAD were also recently reported, [4], [20] which suggests that the drug resistance pattern of *C. difficile* may be changing.

CDAD have been reported in Asia countries such as Japan, Korea, Singapore and Thailand. [21], [22], [23], [24] In Taiwan, the incidence of CDAD has recently been reported as 45 cases per100,000 patient-days, and was highest in medical intensive care units. [25] Few systematic investigations have monitored the drug resistance pattern, prevalence of toxin genes, and bacterial strain clonality in clinical isolates.

Between 2002 and 2007, a total of 2,471 stool specimens were ordered for *C. difficile* cultures at Chang Gung Memorial Hospital, a 3 500-bed medical center in northern Taiwan. A total of 232 non-repeated *C. difficile* isolates from different patients were identified in the clinical microbiology laboratory. Of the 232 isolates, a total of 181 (78%) *C. difficile* isolates were retrospectively retrieved from the bacteria bank for toxin gene testing using the PCR amplification method. A total of 110 toxigenic *C. difficile* isolates were identified and subjected to antimicrobial susceptibility testing and genetic relatedness analysis using a multilocus variable-number tandem-repeat analysis. Further characterization of *tcdC* genotypes and toxinotypes was also performed.

## Materials and Methods

### Ethics Statement

The present study aimed to characterize *C. difficile* isolates using molecular methods. All isolates studied were retrieved retrospectively from the Bacteria Bank, Department of Laboratory Medicine, Chang Gung Memorial Hospital, Linkou. The clinical information of the patients was neither available nor required in this study. The, patient’s informed consent was not required or collected because all microbial cultures were ordered by physicians due to the necessity of clinical management (none were collected purposely for this study). The design and procedure of the study had been approved by the Institutional Review Board of the Chang Gung Memorial Hospital, Linkou, in January 2009.

### Setting

Chang Gung Memorial Hospital (CGMH) is a 3 500-bed university-affiliated medical centre in northern Taiwan. There are 26 intensive care units (ICUs) that are grouped as Medical ICUs, Surgical ICUs, and Pediatric ICUs. The other 73 general wards are included in the Inpatient Department. The Clinical Microbiology Laboratory in the Department of Laboratory Medicine provides routine service for the isolation, identification and antimicrobial susceptibility testing of microbiological pathogens for the entire hospital.

### Bacterial Isolation and Identification

Between 2002 and 2007, a total of 110 non-repeat toxigenic *C. difficile* isolates from different patients were retrospectively retrieved from the bacteria bank for use in the present study. *Clostridium difficile* selective agar (Becton Dickinson, USA) was used for bacterial isolation. Isolates were identified using conventional physiological and biochemical tests and were confirmed using the rapid ID 32A system (BioMerieux, France). All specimens subjected to microbial cultures were ordered by physicians for clinical management.

### Detection of *Tcda*, *Tcdb*, *cdtA*, and *cdtb* Genes

DNA extraction was performed using the QIAamp DNA Mini Kit (Qiagen, Hilden, Germany) according to the manufacturer’s instructions. To determine the presence of toxin genes *tcdA* and *tcdB* in the *C. difficile* isolates, PCR amplification was performed, as previously described. [12], [26] Briefly, two primer pairs, NK9/NK11 and NK104/NK105, were used to amplify the repeating domain of the *tcdA* gene and the non-repeating domain of the *tcdB* gene, respectively. PCR amplification produced the intact *tcdA* gene from A^+^B^+^ isolates yielded a 1,200 bp DNA product. In comparison, shorter DNA fragments of 500 or 700 bp were amplified from the A^−^B^+^ isolates. The binary toxin genes *cdtA* and *cdtB* were detected concurrently using PCR. [12] The primer pairs cdtApos/cdtArev and cdtBpos/cdtBrev were used to amplify a 375-bp fragment from *cdtA*, and a 510-bp fragment from *cdtB*, respectively. The amplified DNA products were separated by agarose gel electrophoresis and photographed under BioDoc-It system (UVP, USA).

### Toxinotyping

The toxigenic *C. difficile* isolates were further characterized using toxinotyping according to the method of Rupnik *et al*. [27] Toxinotyping analyzed the restriction-fragment-length polymorphisms (RFLPs) of the genes encoding toxins A (*tcdA*) and B (*tcdB*) in a region of the *C. difficile* genome known as the pathogenicity locus (PaLoc). We used RFLP analysis for PCR fragments A3 and B1 because this typing assay can identify most of the toxinotypes, [27] in this study.

### PCR Amplification and DNA Sequencing of *tcdc* Gene

To further investigate the *tcdC* gene, the toxigenic *C. difficile* isolates were analyzed using PCR with primers C1 and C2, as previously described. [28] A 718-bp fragment of the PaLoc encompassing the entire *tcdC* gene was amplified. PCR products were purified and subjected to sequencing with amplification (C1 and C2) primers from both directions using a 3100-Avant Genetic Analyzer (Applied Biosystems, USA). The sequences were analyzed and the amino acid sequences deduced using the Lasergene 7.0.0 software package (DNASTAR, Wisconsin, USA) were compared to the wild-type *tcdC* sequence from strain VPI10463 (GenBank accession number Y10689).

### Genotyping by Multilocus Variable-number Tandem-repeat Analysis

The genetic relatedness of the toxigenic *C. difficle* clinical isolates was investigated using multilocus variable-number tandem-repeat analysis (MLVA). MLVA was performed using seven *C. difficile* markers A6*_Cd_*, B7*_Cd_*, C6*_Cd_*, E7*_Cd_*, F3*_Cd_*, G8*_Cd_*, and H9*_Cd_* as previously described, [29] with some modifications. Briefly, genomic *C. difficile* DNA was isolated using the QIAamp DNA Mini Kit (Qiagen, Hilden, Germany) according to the manufacturer’s instructions. The repeats were amplified with respective primer-pair using a single PCR protocol. The amplification reactions were performed in a 25- µl final volume containing 1×PCR buffer, 0.2 mM of each deoxynucleoside triphsphate (GeneTeks BioScience, Taipei, Taiwan), 1 µM of each primer, 0.5 unit HotStar *Taq* DNA polymerase (Qiagen, Hilden, Germany), and 2.5 µl of DNA. An initial denaturation step at 95°C for 15 min was followed by 35 cycles of denaturation at 95°C for 30 sec, primer annealing at 52°C for 30 sec, and extension at 72°C for 30 sec. A final extension step at 72°C for 10 min was added, and the product was stored at 4°C until used. PCR fragments were analyzed using the QIAxcel DNA screening kit (Qiagen) on an HAD-GT12 eGene capillary electrophoresis system (Qiagen, Hilden, Germany) with an internal QX DNA size marker 15 bp-3 kb (Qiagen, Hilden, Germany). The size of each *C. difficile* marker was determined using the software supplied for the electrophoresis apparatus. To verify accurate repeat number assignment, each marker from a selected number of isolates was sequenced. Repeat numbers at each of the seven *C. difficile* markers were concatenated to generate an MLVA type for each isolates. Repeat numbers per locus were entered into BioNumerics software v6.0 (Applied Maths, Texas, USA) for cluster analysis. A dendrogram was constructed using the unweighted-pair group method with arithmetic mean clustering (UPGMA), using the Pearson correlation coefficient.

### Antimicrobial Susceptibility

The minimum inhibitory concentration (MIC) of the 110 toxigenic *C. difficile* isolates against metronidazole and vancomycin was determined using the standard agar dilution method according to the Clinical and Laboratory Standards Institute (CLSI) guidelines. [30] The antibiotics and concentrations used were as followed: 0.25–16 mg/L for vancomycin and 0.5–32 mg/L for metronidazole. The breakpoints for vancomycin and metronidazole were: susceptible, ≦2 mg/L; resistance, >2 mg/L, according to the EUCAST breakpoints. [31].

### Statistics Method

Statistical analyses were performed using Stata (version 11) software (StataCorp LP, USA). The annual number of various toxigenic *C. difficile* isolates by year was analyzed using the Cochran-Armitage test for trend. A *P* value of <0.05 was considered statistically significant.

## Results

### Annual Numbers and Proportions of Various Toxigenic *C. Difficile* Isolates

**Figure 1:**
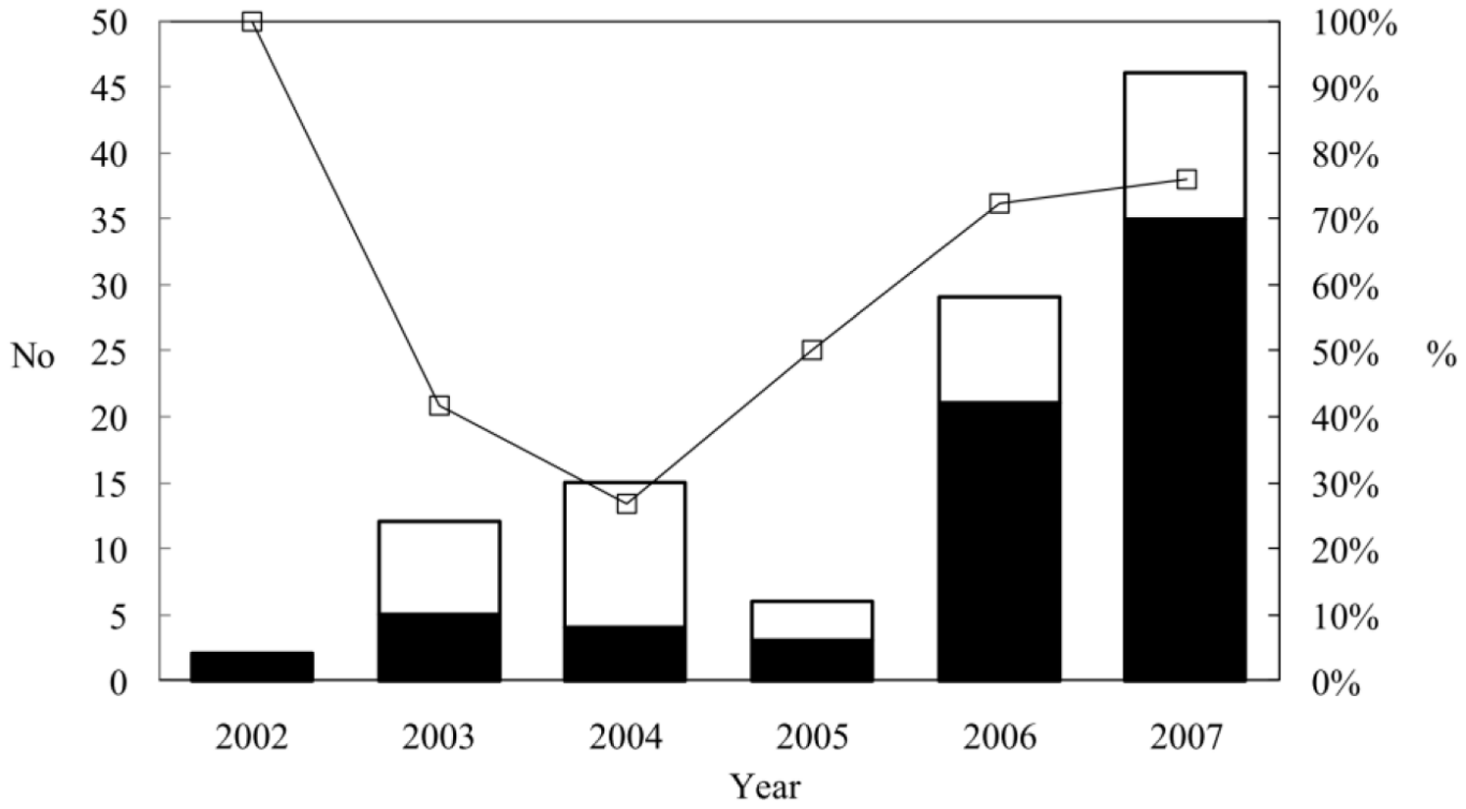
Annual numbers and proportions of various toxigenic *C. difficile* isolates. Solid bars, number of A^+^B^+^ isolates; empty bars, number of A^−^B^+^ isolates; ≤, proportion (%) of A^+^B^+^ isolates.

A total of 110 non-toxigenic *C. difficile* isolates from 110 different patients were studied. Of the 110 patients, 46 were female and 64 were male. Patient ages ranged from four months to 92 years old. Eleven (10%) patients were under the age of 10, and 81 (74%) patients were older than 60 years of age. Of the 110 toxigenic *C. difficile* isolates tested, 70 isolates harbored *tcdA* and *tcdB* (A^+^B^+^) and 40 isolates harbored *tcdB* only (A^−^B^+^). The relationship of coexistence between the toxin genes *tcdA* and *tcdB* and the binary toxin genes *cdtA* and *cdtB* was described in Table 1. Among these, 4 (3.6%) isolates were positive for all four genes (Table 1). The annual numbers and proportions of the 110 toxigenic isolates during the period between 2002 and 2007 are shown in Fig. 1. In 2002, two toxigenic *C. difficile* isolates were A^+^B^+^ isolates. The A^−^B^+^ isolates were first detected in 2003 and accounted for 41.7% of the toxigenic *C. difficile* isolates. In 2004, the proportion of A^−^B^+^ isolates reached a maximum rate of 73.3%, and then decreased to 50% in 2005, dropping to a minimum rate of 23.9% in 2007. In contrast, the proportion of A^+^B^+^ isolates decreased to its lowest level (26.7%) in 2004 and increased to 76.1% in 2007. The annual number of A^+^B^+^ isolates considerably increased over the 6-year study (*P* = 0.055).

**Table 1:** Numbers and types of *C. difficile* isolates determined using toxinotyping, *tcdC* genotyping and the occurrence of toxin genes A, B and the binary toxin genes CDT.

Toxin production type	Isolate no.	Toxinotype(no.)	*tcdC* genotype (no.)
A^−^B^+^CDT^-^	40	VIII (40)	*tcdC-sc7* (40)
A^+^B^+^CDT^-^	66	I (66)	*tcdC-sc0* (16)
			*tcdC-sc3* (2)
			*tcdC-sc9* (47)
			*tcdC-sc15* (1)
A^+^B^+^CDT^+^	4	III (2)	*tcdC-sc1* (2)
		V (2)	*tcdC-A* (2)

A^−^B^+^CDT^-^: toxin A-negative, toxin B-positive, and binary toxin genes-negative *C. difficile*; A^+^B^+^CDT^-^: toxin A-positive, toxin B-positive, and binary toxin genes-negative *C. difficile*; A^+^B^+^CDT^+^: toxin A-positive, toxin B-positive, and binary toxin genes-positive *C. difficile*.

### Toxinotypes and *tcdc* Genotypes of *C. Difficile* Isolates

As shown in Table 1, four different toxinotypes were identified among the 110 toxigenic *C. difficile* isolates, including types I (66 isolates, 60%), VIII (40 isolates, 36.4%), III (2 isolates, 1.8%), and V (2 isolates, 1.8%). Seven previously described *tcdC* types were identified including *tcdC-0* (16 isolates, 14.5%), *tcdC-A* (2 isolates, 1.8%), *tcdC-sc1* (2 isolates, 1.8%), *tcdC-sc3* (2 isolates, 1.8%), *tcdC-sc7* (40 isolates, 36.4%), *tcdC-sc9* (47 isolates, 42.7%), and *tcdC-sc15* (1 isolate, 0.9%). All sequences were identical to sequences deposited in the GenBank, including *tcdC-0* (Y10689), *tcdC-A* (EF470292), *tcdC-sc1* (DQ861412), *tcdC-sc3* (DQ861413), *tcdC-sc7* (DQ861416), *tcdC-sc9* (DQ861418), and *tcdC-sc15* (DQ861423). Moreover, all 40 A^−^B^+^CDT^-^ isolates belonged to toxinotype VIII and *tcdC-sc7*. One toxinotyoe (Toxinotype I) and four different *tcdC* genotypes were identified in the 66 A^+^B^+^CDT^-^ isolates. Two A^+^B^+^CDT^+^ isolates belonged to toxinotype V/*tcdC-A*, and two other A^+^B^+^CDT^+^ isolates belonged to toxinotype III/*tcdC-sc1* (Table 1).

### Resistance Pattern of *C. Difficile* Toxin-producing Strains to Metronidazole and Vancomycin

The MICs of the 110 toxigenic *C. difficile* isolates against the antibiotics metronidazole and vancomycin were determined. The MIC range for metronidazloe was 0.5 to >32 mg/L and 0.25 to >16 mg/L for vancomycin. As shown in Table 2, most isolates were sensitive to both antibiotics. However, two (1.8%) isolates identified in 2003 showed a high resistance to metronidazole (MIC >32 mg/L). Five (4.5%) isolates obtained in 2003 (1 isolate), 2006 (1 isolate) and 2007 (3 isolates), respectively, showed resistance to vancomycin (MIC >2 mg/L). The MIC_90_ for metronidazole and vancomycin were 0.5 and 1 mg/L, respectively, throughout the study period from 2002 to 2007. No isolates were found to be resistant to both drugs.

**Table 2:** Range of MIC values and resistance rate of the 110 toxigenic *C. difficile* isolates analyzed by year.

		Metronidazole				Vancomycin			
Year	No. ofisolates	MIC range(mg/L)	MIC_50_(mg/L)	MIC_90_(mg/L)	Resistance^a^(%)	MIC range(mg/L)	MIC_50_(mg/L)	MIC_90_(mg/L)	Resistance^a^(%)
2002	2	0.5	0.5	0.5	0	0.5	0.5	0.5	0
2003	12	0.5–>32	0.5	>32	2 (16.7)	0.25–8	0.5	2	1 (8.3%)
2004	15	0.5	0.5	0.5	0	0.25–1	0.5	0.5	0
2005	6	0.5	0.5	0.5	0	0.5–1	0.5	1	0
2006	29	0.5	0.5	0.5	0	0.25–16	0.5	1	1 (3.4)
2007	46	0.5–2	0.5	0.5	0	0.25–>16	0.5	2	3 (6.5)
Total	110	0.5–>32	0.5	0.5	2 (1.8)	0.25–>16	0.5	1	5 (4.5)

MIC: minimum inhibitory concentrations.

a The breakpoints for metronidazole and vancomycin recommended by the EUCAST: susceptible, ≦2 mg/L; resistant, >2 mg/L.

### Clonality of *C. Difficile* Isolates

**Figure 2:**
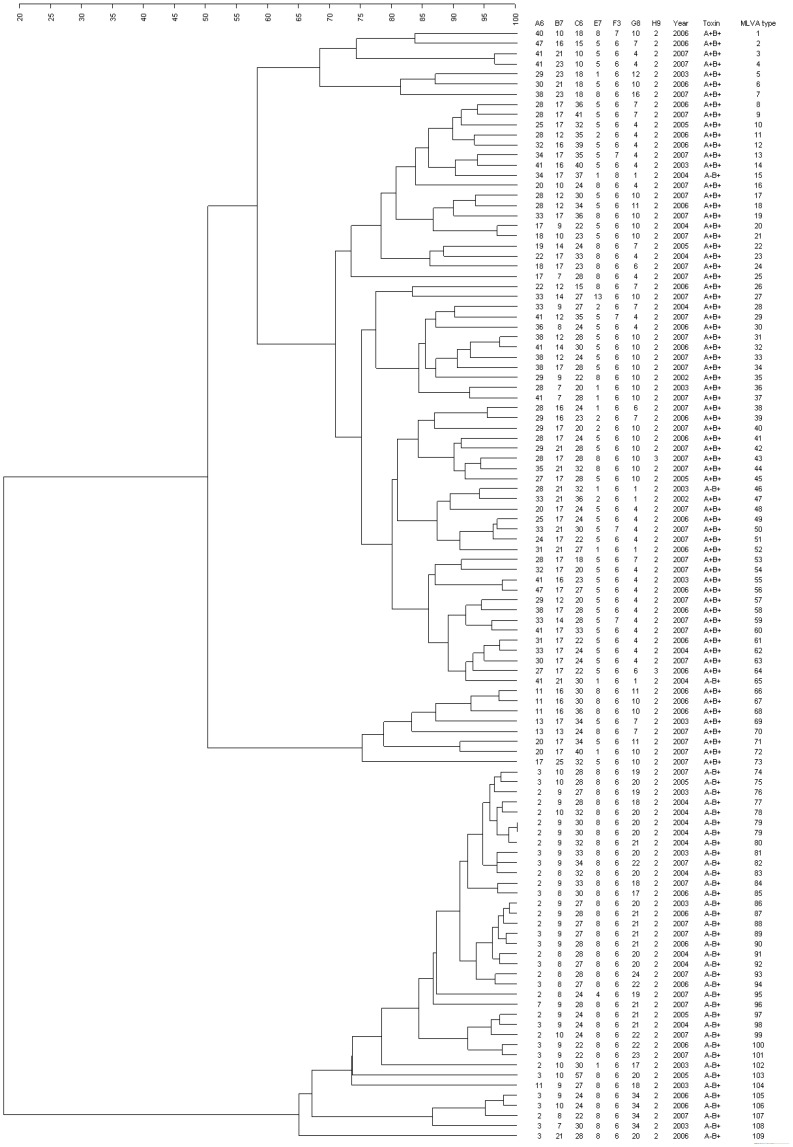
MLVA results of toxigenic *C. difficile* isolates.

To address whether the increasing number is due to clonal spreading of *C. difficile* isolates, the MLVA types of the 110 toxigenic *C. difficile* isolates were analyzed. A total of 109 different MLVA types were identified, in which A^+^B^+^ isolates and A^−^B^+^ isolates were differentiated into two clusters with a similarity of 17.6% (Figure 2). When isolates with 85% similarity were put into MLVA group, 22 different groups were identified among the 70 A^+^B^+^ isolates, while 10 different MLVA groups were found among the 40 A^−^B^+^ isolates (Table 3). Among the A^−^B^+^ *C. difficile* isolates, a major MLVA group (group 1) was identified in 24 (60%) isolates and persisted throughout the study period. The highest numbers were identified in 2004 (8 isolates) and 2007 (8 isolates). In contrast, diverse MLVA types were identified in the 70 A^+^B^+^ isolates, three major MLVA groups (groups 1, 2, and 3) were identified and accounted for 27 isolates (38%) (Table 3).

**Table 3:** MLVA results of the 110 toxigenic *C. difficile* isolates analyzed by year.

			Number of isolate in different year					
Toxin type	MLVA group^a^	Isolate no. (%)	2002	2003	2004	2005	2006	2007
**A^+^B^+^**		**70**	**2**	**5**	**4**	**3**	**21**	**35**
	1	12 (17%)		1	1		4	6
	2	8 (11%)	1		1		2	4
	3	7 (10%)		1		1	3	2
	4	6	1				2	3
	5	5			1		1	3
	6	5				1	1	3
	7	4		1			3	
	8	3			1	1		1
	9	3					1	2
	10	2						2
	11	2		1			1	
	12	2		1				1
	13	2						2
	14	1					1	
	15	1					1	
	16	1						1
	17	1						1
	18	1						1
	19	1					1	
	20	1						1
	21	1						1
	22	1						1
**A** ^−^ **B^+^**		**40**	**0**	**7**	**11**	**3**	**8**	**11**
	1	24 (60%)		3	8	1	4	8
	2	5 (13%)			1	1	1	2
	3	4 (10%)		1			2	1
	4	1		1				
	5	1		1				
	6	1		1				
	7	1			1			
	8	1			1			
	9	1				1		
	10	1					1	

MLVA: multilocus variable-number tandem-repeat analysis.

a *C. difficile* isolates with similarity ≥85% were considered to be a MLVA group.

Regarding to the four A^+^B^+^CDT^+^ isolates, two toxinotype V/*tcdC-A* isolates were genetic related and had a similarity of 97% (MLVA types 66 and 67); these isolates were identified in 2006 (Fig. 2). In contrast, two toxinotype III/*tcdC-sc1* isolates identified in 2007 were genetically unrelated and belonged to MLVA types 15 and 27 (Fig. 2). All the four isolates were found to be genetically different to the hypervirulent ribotype 027 and ribotype 078 isolates in comparison with the MLVA results described previously [32], [33]. Regarding the seven resistant isolates, the MLVA results indicated that the two metronidazole-resistant A^+^B^+^ isolates identified in 2003 were genetically unrelated (MLVA types 14 and 55). In contrast, there were five vancomycin-resistant isolates, including three A^+^B^+^ isolates identified in 2007 and two A^−^B^+^ isolates identified in 2003 and 2006. Two of the three vancomycin-resistant A^+^B^+^ isolates were closely related and had a similarity of 94% (MLVA types 43 and 44). The other one A^+^B^+^ isolate and two A^−^B^+^ isolates were genetically unrelated (MLVA types 31, 102 and 109).

## Discussion

The proportions of the 110 toxigenic *C. difficile* isolates over the 6-years differed from year to year, and an increasing number of A^+^B^+^ isolates was observed over this period (Fig. 1). Further MLVA analysis of these toxigenic *C. difficile* isolates showed that an increasing number of genetic divergences between the *C. difficile* A^+^B^+^ isolates (Table 3). In addition, the percentage of A^−^B^+^ isolates reached 73.3% in 2004. One major MLVA group (MLVA-group 1) accounted for 60% of the 40 A^−^B^+^ isolates. A clonal dissemination of the A^−^B^+^ isolates similar to the outbreaks identified in Japan, Korea, Canada, and Poland was identified in our hospital. [34], [35], [36], [37] After 2004, the proportion of A^−^B^+^ isolates began to decrease and dropped to 23.9% in 2007. *C. difficile* A^−^B^+^ isolates appeared to be an endemic strain to our hospital and is worthy of closely monitoring.

Characterization of the phylogenic relatedness of the *C. difficile* isolates by toxinotypes was achieved using PCR-RFLP for PaLoc and by directly DNA sequencing the *tcdC* gene. The results from both methods were essentially concordant. Of the studied isolates, toxinotypes III, V, and VIII were consistently associated with *tcdC* genotypes *tcdC-sc1*, *tcdC-A* and, *tcdC-sc7*, respectively (Table 1). In this study, all A-B+ isolates belonged to toxinotype VIII and *tcdC-sc7* genotypes. The results were similar to those of previous reports. [38], [39] Earlier reports indicated that the *tcdC* gene involves the negative regulation of *tcdA* and *tcdB* expression. [5] Many *C. difficile* isolates defective in *tcdC* were reported and grouped as ribotype 027 and toxinotype III. For example, the *tcdC-A* genotype which contains a nonsense mutation (C184T) and a 39-bp deletion from nucleotides 341 to 379, encodes a truncated 61-amino-acid TcdC protein. [28] Additionally, the *tcdC-sc1* genotype, which contains a single deletion of nucleotide A117 and an additional 18-bp deletion from nucleotides 330 to 347, produces a truncated 65-amino-acid TcdC protein. [40] These two strains with truncated *tcdC* generated nonfunctional TcdC and are responsible for the increased toxin production and virulence of *C. difficile* strains. [40] However, several studies reported contradictory results. Previous reports indicated that deletion or truncation of the *tcdC* gene was often found in toxigenic *C. difficile* but lacked association with disease severity. [41], [42] In the present study, four isolates carrying all four *tcdA*, *tcdB*, *cdtA* and *cdtB* genes also harbored a 18-bp deletion (*tcdC-sc1*) or a 39-bp deletion (*tcdC-A*) (Table 1). However, no serious clinical symptoms were observed in the four patients (data not shown). Because the number of such isolates was too low to make any suggestions or conclusions, the relationship between the *tcdC* deletions and the development of more severe *C. difficile* diseases still awaits further investigation. In addition, the four isolates are genetically different to the hypervirulent ribotype 027 and ribotype 078 strains in comparison with the MLVA results of ribotype 027 and ribotype 078 strains from published references. [32], [33] (data not shown) It appeared that there remains no evidence for the existence of the hypervirulent NAP1/027 strain in Taiwan.

Antimicrobial susceptibility testing identified seven resistant isolates (6.4%) including two metronidazole-resistant and five vancomycin-resistant isolates. Most isolates represented sporadic case, with the exception of two genetically related vancomycin-resistant A^+^B^+^ isolates indentified in 2007. Although the percentage of drug resistance among the *C. difficile* isolates was not high compared to previous reports, [19] emerging drug resistance in the toxin-producing isolates, especially in the increasing A^+^B^+^ isolates, warrants concern. However, because the isolates for antimicrobial susceptibility testing were retrospectively retrieved from the bacteria bank, some metronidazole-heteroresistant populations may not be detectable using the agar dilution method. [19] Therefore, the number of metronidazole-resistant isolates may be underestimated.

In conclusion, the changing trend of various toxigenic *C. difficile* isolates was studied. Results indicated a persistence of MLVA group 1 A^−^B^+^ isolates and an increase of A^+^B^+^ isolates with diverse MLVA types between 2002 and 2007. Some *C. difficile* isolates with antimicrobial resistance to metronidazole or vancomycin have been identified. Continuous monitor is warranted to understand the developing situation and to control the further spread of such infections, especially among hospitalized patients.

## References

[1] Kelly CP, Pothoulakis C, LaMont JT (1994) *Clostridium difficile* colitis. N Engl J Med 330: 257–262. 8043060 10.1056/NEJM199401273300406

[2] McDonald LC, Killgore GE, Thompson A, Owens RC Jr, Kazakova SV, et al (2005) An epidemic, toxin gene-variant strain of *Clostridium difficile* . N Engl J Med 353: 2433–2441. 16322603 10.1056/NEJMoa051590

[3] Warny M, Pepin J, Fang A, Killgore G, Thompson A, et al (2005) Toxin production by an emerging strain of *Clostridium difficile* associated with outbreaks of severe disease in North America and Europe. Lancet 366: 1079–1084. 16182895 10.1016/S0140-6736(05)67420-X

[4] Kelly CP, LaMont JT (2008) *Clostridium difficile*-more difficult than ever. N Engl J Med 359: 1932–1940. 18971494 10.1056/NEJMra0707500

[5] Voth DE, Ballard JD (2005) *Clostridium difficile* toxins: mechanism of action and role in disease. Clin Microbiol Rev 18: 247–263. 15831824 10.1128/CMR.18.2.247-263.2005 PMC1082799

[6] Kuehne SA, Cartman ST, Heap JT, Kelly ML, Cockayne A, et al (2010) The role of toxin A and toxin B in *Clostridium difficile* infection. Nature 467: 711–713. 20844489 10.1038/nature09397

[7] Geric B, Carman RJ, Rupnik M, Genheimer CW, Sambol SP, et al (2006) Binary toxin-producing, large clostridial toxin-negative *Clostridium difficile* strains are enterotoxic but do not cause disease in hamsters. J Infect Dis 193: 1143–1150. 16544255 10.1086/501368

[8] Sundriyal A, Roberts AK, Ling R, McGlashan J, Shone CC, et al. (2010) Expression, purification and cell cytotoxicity of actin-modifying binary toxin from *Clostridium difficile*. Protein Expr Purif. 10.1016/j.pep.2010.04.014 20433927

[9] Elliott B, Reed R, Chang BJ, Riley TV (2009) Bacteremia with a large clostridial toxin-negative, binary toxin-positive strain of *Clostridium difficile* . Anaerobe 15: 249–251. 19723585 10.1016/j.anaerobe.2009.08.006

[10] Wilkins TD, Lyerly DM (2003) *Clostridium difficile* testing: after 20 years, still challenging. J Clin Microbiol 41: 531–534. 12574241 10.1128/JCM.41.2.531-534.2003 PMC149726

[11] Knoop FC, Owens M, Crocker IC (1993) *Clostridium difficile*: clinical disease and diagnosis. Clin Microbiol Rev 6: 251–265. 8358706 10.1128/cmr.6.3.251 PMC358285

[12] Terhes G, Urban E, Soki J, Hamid KA, Nagy E (2004) Community-acquired *Clostridium difficile* diarrhea caused by binary toxin, toxin A, and toxin B gene-positive isolates in Hungary. J Clin Microbiol 42: 4316–4318. 15365032 10.1128/JCM.42.9.4316-4318.2004 PMC516352

[13] Persson S, Torpdahl M, Olsen KE (2008) New multiplex PCR method for the detection of *Clostridium difficile* toxin A (tcdA) and toxin B (tcdB) and the binary toxin (cdtA/cdtB) genes applied to a Danish strain collection. Clin Microbiol Infect 14: 1057–1064. 19040478 10.1111/j.1469-0691.2008.02092.x

[14] Wroblewski D, Hannett GE, Bopp DJ, Dumyati GK, Halse TA, et al (2009) Rapid molecular characterization of *Clostridium difficile* and assessment of populations of *C. difficile* in stool specimens. J Clin Microbiol 47: 2142–2148. 19403775 10.1128/JCM.02498-08 PMC2708487

[15] Peterson LR, Manson RU, Paule SM, Hacek DM, Robicsek A, et al (2007) Detection of toxigenic *Clostridium difficile* in stool samples by real-time polymerase chain reaction for the diagnosis of *C. difficile*-associated diarrhea. Clin Infect Dis 45: 1152–1160. 17918076 10.1086/522185

[16] Larson AM, Fung AM, Fang FC (2010) Evaluation of t*cdB* real-time PCR in a three-step diagnostic algorithm for detection of toxigenic *Clostridium difficile* . J Clin Microbiol 48: 124–130. 19923482 10.1128/JCM.00734-09 PMC2812255

[17] Cohen SH, Gerding DN, Johnson S, Kelly CP, Loo VG, et al (2010) Clinical practice guidelines for *Clostridium difficile* infection in adults: 2010 update by the society for healthcare epidemiology of America (SHEA) and the infectious diseases society of America (IDSA). Infect Control Hosp Epidemiol 31: 431–455. 20307191 10.1086/651706

[18] Huang H, Weintraub A, Fang H, Nord CE (2009) Antimicrobial resistance in *Clostridium difficile* . Int J Antimicrob Agents 34: 516–522. 19828299 10.1016/j.ijantimicag.2009.09.012

[19] Pelaez T, Alcala L, Alonso R, Rodriguez-Creixems M, Garcia-Lechuz JM, et al (2002) Reassessment of *Clostridium difficile* susceptibility to metronidazole and vancomycin. Antimicrob Agents Chemother 46: 1647–1650. 12019070 10.1128/AAC.46.6.1647-1650.2002 PMC127235

[20] Shah D, Dang MD, Hasbun R, Koo HL, Jiang ZD, et al (2010) *Clostridium difficile* infection: update on emerging antibiotic treatment options and antibiotic resistance. Expert Rev Anti Infect Ther 8: 555–564. 20455684 10.1586/eri.10.28 PMC3138198

[21] Koh TH, Tan AL, Tan ML, Wang G, Song KP (2007) Epidemiology of *Clostridium difficile* infection in a large teaching hospital in Singapore. Pathology 39: 438–442. 17676487 10.1080/00313020701444507 PMC7130798

[22] Pupaibool J, Khantipong M, Suankratay C (2008) A study of *Clostridium difficile*-associated disease at King Chulalongkorn Memorial Hospital, Thailand. J Med Assoc Thai 91: 37–43. 18386542

[23] Sawabe E, Kato H, Osawa K, Chida T, Tojo N, et al (2007) Molecular analysis of *Clostridium difficile* at a university teaching hospital in Japan: a shift in the predominant type over a five-year period. Eur J Clin Microbiol Infect Dis 26: 695–703. 17647032 10.1007/s10096-007-0355-8

[24] Shin BM, Kuak EY, Yoo HM, Kim EC, Lee K, et al (2008) Multicentre study of the prevalence of toxigenic *Clostridium difficile* in Korea: results of a retrospective study 2000–2005. J Med Microbiol 57: 697–701. 18480325 10.1099/jmm.0.47771-0

[25] Lee YC, Wang JT, Chen AC, Sheng WH, Chang SC, et al. (2011) Changing incidence and clinical manifestations of *Clostridium difficile*-associated diarrhea detected by combination of glutamate dehydrogenase and toxin assay in Northern Taiwan. J Microbiol Immunol Infect. 10.1016/j.jmii.2011.12.001 22209696

[26] Kato H, Kato N, Watanabe K, Iwai N, Nakamura H, et al (1998) Identification of toxin A-negative, toxin B-positive *Clostridium difficile* by PCR. J Clin Microbiol 36: 2178–2182. 9665986 10.1128/jcm.36.8.2178-2182.1998 PMC105000

[27] Rupnik M, Avesani V, Janc M, von Eichel-Streiber C, Delmee M (1998) A novel toxinotyping scheme and correlation of toxinotypes with serogroups of *Clostridium difficile* isolates. J Clin Microbiol 36: 2240–2247. 9665999 10.1128/jcm.36.8.2240-2247.1998 PMC105025

[28] Spigaglia P, Mastrantonio P (2002) Molecular analysis of the pathogenicity locus and polymorphism in the putative negative regulator of toxin production (TcdC) among *Clostridium difficile* clinical isolates. J Clin Microbiol 40: 3470–3475. 12202595 10.1128/JCM.40.9.3470-3475.2002 PMC130716

[29] van den Berg RJ, Schaap I, Templeton KE, Klaassen CH, Kuijper EJ (2007) Typing and subtyping of *Clostridium difficile* isolates by using multiple-locus variable-number tandem-repeat analysis. J Clin Microbiol 45: 1024–1028. 17166961 10.1128/JCM.02023-06 PMC1829118

[30] Clinical and Laboratory Standards Institute (2007) Methods for antimicrobial susceptibility testing of anaerobic bacteria 7th ed. Clinial Laboratory and Standards Institute, Wayne, Pa.

[31] European Committee on Antimicrobial Susceptibility Testing (2013) Breakpoint tables for interpretation of MICs and zone diameters. Version 3.1. Available: http://www.eucast.org/fileadmin/src/media/PDFs/EUCAST_files/Breakpoint_tables/Breakpoint_table_v_3.1.pdf. Accessed 2013 Feb 20.

[32] Fawley WN, Freeman J, Smith C, Harmanus C, van den Berg RJ, et al (2008) Use of highly discriminatory fingerprinting to analyze clusters of *Clostridium difficile* infection cases due to epidemic ribotype 027 strains. J Clin Microbiol 46: 954–960. 18216211 10.1128/JCM.01764-07 PMC2268363

[33] Bakker D, Corver J, Harmanus C, Goorhuis A, Keessen EC, et al (2010) Relatedness of human and animal Clostridium difficile PCR ribotype 078 isolates determined on the basis of multiplelocus variable-number tandem-repeat analysis and tetracycline resistance. J Clin Microbiol 48: 3744–3749. 20686080 10.1128/JCM.01171-10 PMC2953124

[34] Kim H, Riley TV, Kim M, Kim CK, Yong D, et al (2008) Increasing prevalence of toxin A-negative, toxin B-positive isolates of *Clostridium difficile* in Korea: impact on laboratory diagnosis. J Clin Microbiol 46: 1116–1117. 18199783 10.1128/JCM.01188-07 PMC2268382

[35] Komatsu M, Kato H, Aihara M, Shimakawa K, Iwasaki M, et al (2003) High frequency of antibiotic-associated diarrhea due to toxin A-negative, toxin B-positive *Clostridium difficile* in a hospital in Japan and risk factors for infection. Eur J Clin Microbiol Infect Dis 22: 525–529. 12938013 10.1007/s10096-003-0992-5

[36] al-Barrak A, Embil J, Dyck B, Olekson K, Nicoll D, et al (1999) An outbreak of toxin A negative, toxin B positive *Clostridium difficile*-associated diarrhea in a Canadian tertiary-care hospital. Can Commun Dis Rep 25: 65–69. 10344088

[37] Pituch H, van den Braak N, van Leeuwen W, van Belkum A, Martirosian G, et al (2001) Clonal dissemination of a toxin-A-negative/toxin-B-positive *Clostridium difficile* strain from patients with antibiotic-associated diarrhea in Poland. Clin Microbiol Infect 7: 442–446. 11591209 10.1046/j.1198-743x.2001.00312.x

[38] Rupnik M (2008) Heterogeneity of large clostridial toxins: importance of *Clostridium difficile* toxinotypes. FEMS Microbiol Rev 32: 541–555. 18397287 10.1111/j.1574-6976.2008.00110.x

[39] Rupnik M, Kato N, Grabnar M, Kato H (2003) New types of toxin A-negative, toxin B-positive strains among *Clostridium difficile* isolates from Asia. J Clin Microbiol 41: 1118–1125. 12624039 10.1128/JCM.41.3.1118-1125.2003 PMC150296

[40] Curry SR, Marsh JW, Muto CA, O’Leary MM, Pasculle AW, et al (2007) *tcdC* genotypes associated with severe TcdC truncation in an epidemic clone and other strains of *Clostridium difficile* . J Clin Microbiol 45: 215–221. 17035492 10.1128/JCM.01599-06 PMC1828959

[41] Verdoorn BP, Orenstein R, Rosenblatt JE, Sloan LM, Schleck CD, et al (2010) High prevalence of tcdC deletion-carrying Clostridium difficile and lack of association with disease severity. Diagn Microbiol and Infec Dis 66: 24–28. 19775847 10.1016/j.diagmicrobio.2009.08.015

[42] Goldenberg SD, French GL (2011) Lack of association of tcdC type and binary toxin status with disease severity and outcome in toxigenic Clostridium difficile. J Infect 62: 355–362. 21396957 10.1016/j.jinf.2011.03.001

